# Distribution Features of Skeletal Metastases: A Comparative Study between Pulmonary and Prostate Cancers

**DOI:** 10.1371/journal.pone.0143437

**Published:** 2015-11-23

**Authors:** Changyin Wang, Ying Shen, Shaobo Zhu

**Affiliations:** 1 Department of Nuclear Medicine, Zhongnan Hospital of Wuhan University, Wuhan Hubei 430071, People’s Republic of China; 2 Department of Osteology, Zhongnan Hospital of Wuhan University, Wuhan Hubei, 430071, People’s Republic of China; University Medical Center Hamburg-Eppendorf, GERMANY

## Abstract

Bone scintigraphies are widely applied for detecting bone metastases. The aim of this study was to investigate distribution features of bone metastases in pulmonary and prostate cancers. Bone scintigraphies were performed in 460 patients with pulmonary cancer and 144 patients with prostate cancer. Patients were divided into three groups according to the total number of bone metastases: few bone metastases, moderate bone metastases, and extensive bone metastases. We compared the distribution of bone metastases in the two cancers, and analyzed the relationship between the distribution of metastatic lesions and their metastatic patterns. A total of 2279 and 2000 lesions of bone metastases were detected in 258 patients with pulmonary cancer and 102 patients with prostate cancer, respectively. In patients with few bone metastases, the distributions of metastatic lesions in the vertebrae (χ^2^ = 16.0, P = 0.000) and thoracic bones (χ^2^ = 20.7, P = 0.002) were significantly different between pulmonary and prostate cancers. In cases with moderate bone metastases, the distributions in the vertebrae (χ^2^ = 6.6, P = 0.010), pelvis (χ^2^ = 15.1 P = 0.000), and thoracic bones (χ^2^ = 38.8, P = 0.000) were also significantly different between the two cancers. However, in patients with extensive bone metastases, the distributions were very similar. As the total number of bone metastases increased, their distribution in pulmonary cancer did not noticeably change, but the distribution in the vertebrae and thoracic bones of prostate cancer patients significantly changed. Accordingly, the distribution characteristics of bone metastases differed in pulmonary and prostate cancers, mainly in the early stages of metastasis.

## Introduction

Pulmonary and prostate cancers are the most common malignant tumors, and often develop into bone metastases. Tumor cells spread to the bones mainly by hematogenous metastasis; however, the mechanisms underlying bone metastasis in pulmonary and prostate cancers are different. Specifically, pulmonary cancer cells metastasize to bones mainly by pulmonary veins, whereas prostate cancer cells spread to bones mainly via the vertebral venous system [1, 2]. The different routes of metastasis can produce different distribution features of bone metastases, although the studies by Morgan et al. [3] and Dodds et al. [4] do not support this viewpoint. Bone scintigraphy is an important technique in the detection of bone metastases for evaluating the stage of the malignant tumor [5] and the prognosis of the patient [6], as well as for assessing the tumor response to therapy [7], and the effectiveness of the therapeutic schedule [8]. This tool can be used to scan the entire skeleton of patients, thereby allowing all bone lesions to be displayed in their entirety. Thus, bone scintigraphy is very useful for studying the distributional features of bone metastases and analyzing the relationship between the pathways of tumor spread and the distribution of metastatic lesions.

There have been numerous studies about bone metastases in pulmonary and prostate cancers, but few of them have been comparative statistical analyses [9, 10]. Many studies have shown that the predilection sites of bone metastases in pulmonary [11–14] and prostate cancers [15–17] are similar and include the vertebrae, pelvis, and ribs. However, there are little data on differences in the distribution of bone metastases between pulmonary and prostate cancers. An understanding of these differences will be helpful for the differential diagnosis of these cancers.

We classified the patients according to the total number of bone metastases. Then, we used lesion-based analysis to investigate the changes in distribution of metastatic bone lesions in cases with different numbers of lesions and to compare the distribution features of bone metastases between pulmonary and prostate cancers. We found that the distribution characteristics of bone metastases differed in these cancer types.

## Materials and Methods

### 1. Clinical Patients

This was a retrospective study. The data were extracted from a picture archiving and communication system (PACS) between January 2007 and April 2011 in Zhongnan Hospital of Wuhan University (Hubei, China). Bone scintigraphies were performed in 460 patients with pulmonary cancer (343 men, 117 women; aged 24–89; median age, 61.0) and 144 male patients with prostate cancer (aged 38–92; median age, 72.5). A definitive diagnosis using pathological techniques was made in all of the patients. For each patient, only results of the whole-body bone scan that was performed before or after diagnosis was confirmed by pathology, were selected for this study. If needed, other follow-up bone scans were used as diagnostic references. This clinical study was not limited to a specific disease stage, and was approved by the Ethics Committees of Zhongnan Hospital. Because the data were analyzed anonymously, consent was waived.

### 2. Imaging Method

Bone scintigraphy was performed using a single-headed gamma camera with a low-energy and high-resolution collimator (E.CAM; Siemens, Hoffman Estates, IL, USA). All of the patients were instructed to drink about 1000 ml water within 2 h after intravenous injection of 740 MBq of ^99m^Tc-methylene diphosphonate (^99m^Tc-MDP), and to empty their urine before bone scintigraphy. All of the planar bone scans, including anterior and posterior projections, were performed approximately 3 h after injection of imaging reagents, according to the following conditions: patients in a supine posture; 256 × 1024 matrix; zoom, 1.0; and movement velocity of examination bed, 15–25 cm/min. If necessary, additional local planar bone imaging and a single photon emission computed tomography (SPECT) scan were performed.

### 3. Image Interpretation and Analysis of Findings

Analyses of planar bone scintigraphy and SPECT bone scans were made as a consensus reading of two nuclear medicine physicians, and those of X-rays, computed tomography (CT), and magnetic resonance imaging (MRI) were made as a consensus reading of a nuclear medicine physician and radiologist.

According to the diagnostic method by Even-Sapir et al. [17], we used the following criteria to diagnose bone metastasis: 1) Bone lesions with increased uptake of ^99m^Tc-MDP were considered bone metastases when vertebral lesions involved the posterior aspect of the vertebral body and pedicle or extensively involved the vertebra [18, 19], when rib lesions appeared elongated [20], when the tumor was lamellar [20], or when bone lesions presented at multiple sites with scrambled arrangement; 2) Focal bone lesions with increased uptake of ^99m^Tc-MDP were regarded as malignant when they were excluded from being benign bone diseases such as fractures; 3) Bone lesions with decreased uptake of ^99m^Tc-MDP were categorized as bone metastases when postradiotherapy changes, metallic influences, and benign bone diseases such as bone cysts were excluded; 4) Bone lesions with abnormal uptake of ^99m^Tc-MDP were deemed malignant when they were simultaneously confirmed as being bone metastases by X-ray, CT, or MRI; 5) By comparing the results of bone scans that were performed at different times, new bone lesions were considered malignant when they could not be diagnosed as benign, or when the bone scan showed the “flare phenomenon” [21, 22].

No bone metastasis was noted in the bone scintigraphies findings under the following circumstances: 1) when no abnormal radiotracer uptake was detected; 2) when there was indeterminate decreased radioactivity in local bones; 3) when bone lesions showed increased uptake of ^99m^Tc-MDP around the joints [17]; and 4) when bone lesions with abnormal radiotracer uptake were characterized as being benign conditions such as bone fracture, bone cyst, hyperosteogeny, osteophyte, bone-bridge, degenerative osteoarthropathy, or inflammation.

### 4. Grouping of Bone Metastases

According to the method of Wilson and Calhoun [10], we fractionated the whole bones into five regions: 1) thoracic bones, including ribs, sternum, collarbone, and bladebone; 2) vertebrae, including cervical spine, thoracic spine, and lumbar spine; 3) pelvis, including sacrococcyx, ilium, ischium, and pubis; 4) skull, including bones of the cerebral cranium and facial cranium; and 5) extremities, including humerus, femur, radioulna, and tibiofibula. To study the relationship between the distribution of bone metastases and the total number of lesions that metastasized to the bones, and to compare the differences in the distribution of bone metastases between pulmonary and prostate cancers, we divided patients into three groups according to the total number of bone metastases: few bone metastases (1–3), moderate bone metastases (4–10), and extensive bone metastases (>10) [23].

### 5. Statistical Analysis

Data were analyzed with SPSS statistical software (version 13.0; SPSS Inc., Chicago, IL, USA). The Chi-square test was performed to compare differences in the proportions of bone metastases between different groups, and between pulmonary and prostate cancers. P values less than 0.05 were considered statistically significant.

## Results

### 1. Total Distribution of Bone Metastases

Of the 604 patients with malignant tumors, 360 had bone metastases and 4279 lesions of bone metastasis were detected. The incidence (70.8%, 102/144) of bone metastasis in prostate cancer was significantly higher than that (56.1%, 258/460) in pulmonary cancer (χ^2^ = 10.2, p = 0.001). Bone metastases in both pulmonary (89.3%, 2035/2279) and prostate cancers (89.5%, 1790/2000) were mainly distributed in the vertebrae, pelvis, and thoracic bones. The results in Table 1 show that the distribution of metastatic lesions in the pelvis (e.g., sacrococcyx, ilium, ischium, pubis, S1 Table) of patients with prostate cancer was slightly greater than that in patients with pulmonary cancer (χ^2^ = 26.5, p = 0.000), whereas the distribution of metastatic lesions in the thoracic bones (mainly ribs and bladebone, S1 Table) of patients with pulmonary cancer was slightly higher than that in prostate cancer patients (χ^2^ = 9.9, p = 0.002). In general, there were no significant differences between pulmonary and prostate cancers with regard to the different bone regions (Table 1).

**Table 1:** Comparison of total distribution of bone metastases (n = 4279) between pulmonary and prostate cancers.

Skeleton	Pulmonary cancer (n = 2279)		Prostate cancer (n = 2000)		*χ* ^2^	*p* value
	n	%	n	%		
**Vertebrae**	633	27.78	516	25.80	2.119	0.146
**Pelvis**	496	21.76	572	28.60	26.537	0.000
**Thoracic bones**	906	39.75	702	35.10	9.852	0.002
**Skull**	81	3.55	65	3.25	0.300	0.584
**Extremities**	163	7.15	145	7.25	0.015	0.902

Note: n, the lesion number of bone metastases. Chi-square test of likelihood ratio was performed to compare the different proportions of bone metastases between pulmonary and prostate cancers.

### 2. Comparison of Bone Metastases between Pulmonary and Prostate Cancers in Patients with Few Bone Metastases

Table 2 summarizes the distribution of metastatic bone lesions in patients with few bone metastases. The proportion of prostate cancer metastasis to the vertebral column was two-fold more than that of pulmonary cancer (χ^2^ = 19.6, p = 0.000), with the lumbar vertebrae accounting for a large part of the difference (S2 Table). The proportion of thoracic bone metastases in pulmonary cancer was approximately four-fold more than that in prostate cancer (χ^2^ = 20.7, p = 0.000), mainly due to differences in the number of metastases in the ribs and bladebone (S2 Table). Differences in the proportion of bone metastases in the pelvis, skull, and extremities between pulmonary and prostate cancers were not significant (p > 0.05). These data show that there is a significantly different distribution of metastatic lesions in the vertebrae and thoracic bones of patients with pulmonary and prostate cancers and with few bone metastases compared to those with moderate or extensive metastases.

**Table 2:** Comparison of bone metastases between pulmonary and prostate cancers in patients with few bone metastases (n = 304).

Skeleton	Pulmonary cancer (n = 246)		Prostate cancer (n = 58)		*χ* ^2^	*p* value
	n	%	n	%		
**Vertebrae**	71	28.86	35	60.34	15.958	0.000
**Pelvis**	47	19.11	14	24.14	0.715	0.398
**Thoracic bones**	97	39.43	6	10.34	20.736	0.000
**Skull**	6	2.44	0	0.00	-	-
**Extremities**	25	10.16	3	5.17	1.584	0.208

Note: n, the lesion number of bone metastases. Chi-square test of likelihood ratio was performed to compare the different proportions of bone metastases between pulmonary and prostate cancers.

### 3. Comparison of Bone Metastases between Pulmonary and Prostate Cancers in Patients with Moderate Bone Metastases

The distribution of bone metastases in pulmonary and prostate cancers in patients with moderate bone metastases are listed in Table 3. The proportion of vertebral metastases in prostate cancer was greater than that in pulmonary cancer (χ^2^ = 6.6, p = 0.010), and the proportion of thoracic bone metastases in pulmonary cancer was more than that in prostate cancer (χ^2^ = 38.8, P = 0.000). However, the increased proportions in patients with moderate bone metastases were lower than those in patients with few bone metastases. Furthermore, the distribution of prostate cancer metastases in the pelvis was significantly more than that of pulmonary cancer (χ^2^ = 15.1, p = 0.000).

**Table 3:** Comparison of bone metastases between pulmonary and prostate cancers in patients with moderate bone metastases (n = 640).

Skeleton	Pulmonary cancer (n = 479)		Prostate cancer (n = 161)		χ^2^	*p* value
	n	%	n	%		
**Vertebrae**	143	29.85	66	40.99	6.640	0.010
**Pelvis**	88	18.37	54	33.54	15.102	0.000
**Thoracic bones**	194	40.50	24	14.91	38.823	0.000
**Skull**	17	3.55	5	3.11	0.073	0.787
**Extremities**	37	7.72	12	7.45	0.013	0.911

Note: n, the lesion number of bone metastases. Chi-square test of likelihood ratio was performed to compare the different proportions of bone metastases between pulmonary and prostate cancers.

### 4. Comparison of Bone Metastases between Pulmonary and Prostate Cancers in Patients with Extensive Bone Metastases

The distribution of metastatic bone lesions in patients with extensive bone metastases is shown in Table 4. The proportion of vertebral metastases in prostate cancer was slightly lower than that in pulmonary cancer (χ^2^ = 0.9, p = 0.015), and the difference in thoracic bone lesions between pulmonary and prostate cancers was not significant (χ^2^ = 1.2, P = 0.275), in contrast to the results in patients with few and moderate bone metastases. The distribution of bone metastases in the pelvis in prostate cancer was slightly more than that in pulmonary cancer (χ^2^ = 11.1, p = 0.001); however, the distribution of metastatic lesions in the skull and extremities was not significantly different between pulmonary and prostate cancers. Thus, the distribution of bone metastases in pulmonary and prostate cancers is very similar in patients with extensive bone metastases.

**Table 4:** Comparison of bone metastases between pulmonary and prostate cancers in patients with extensive bone metastases (n = 3335).

Skeleton	Pulmonary cancer (n = 1554)		Prostate cancer (n = 1781)		*χ* ^2^	*p* value
	n	%	n	%		
**Vertebrae**	419	26.96	415	23.30	5.923	0.015
**Pelvis**	361	23.23	504	28.30	11.144	0.001
**Thoracic bones**	615	39.58	672	37.73	1.190	0.275
**Skull**	58	3.73	60	3.37	0.321	0.571
**Extremities**	101	6.50	130	7.30	0.826	0.363

Note: n, the lesion number of bone metastases. Chi-square test of likelihood ratio was performed to compare the different proportions of bone metastases between pulmonary and prostate cancers.

### 5. Comparison of Bone Metastasis Distribution in Different Lesions

**Fig 1:**
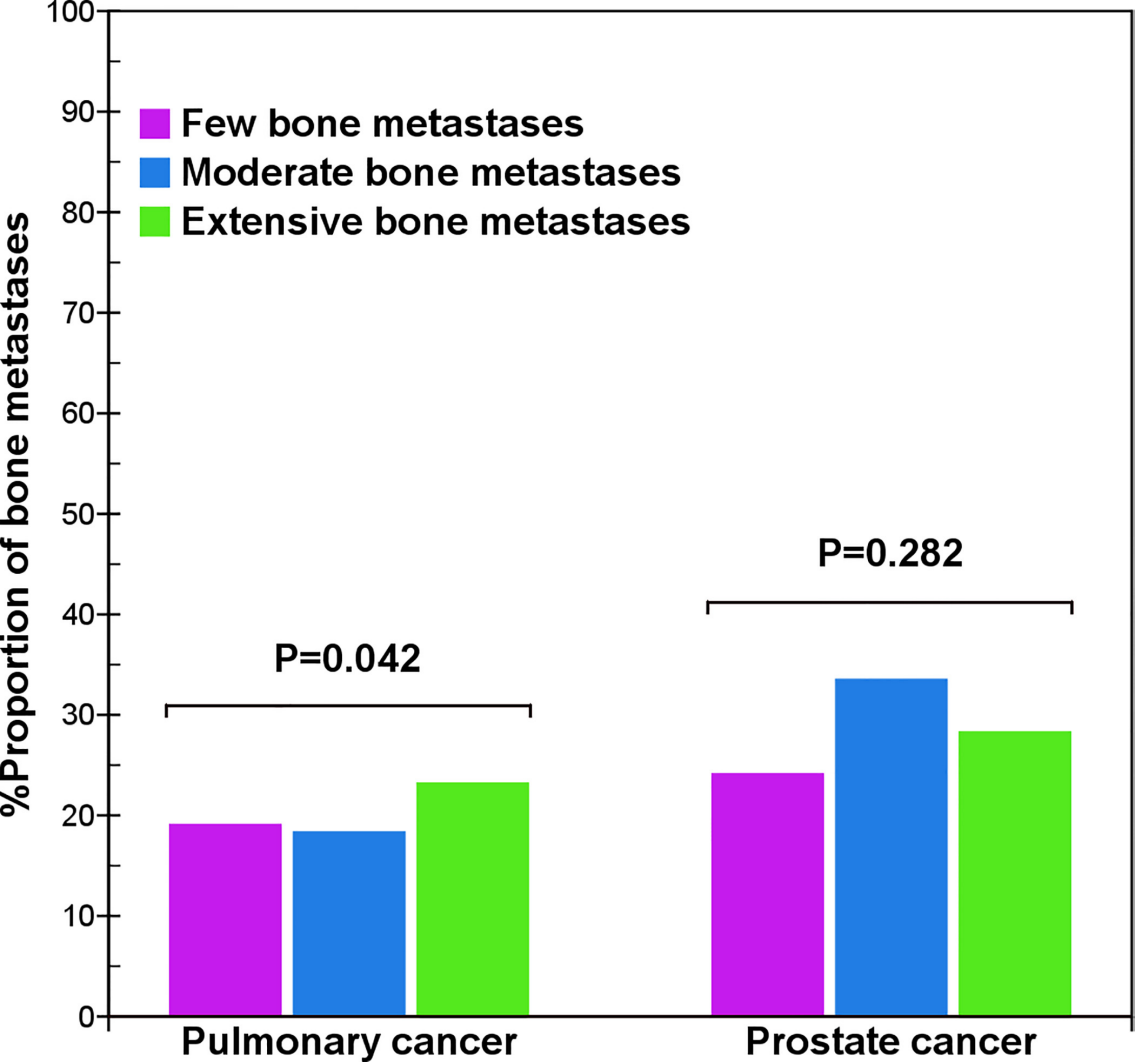
Comparison of pelvis metastases between pulmonary and prostate cancers. When the total number of bone metastases increased, the distribution in the pelvis in pulmonary cancer presents a smaller difference (χ^2^ = 6.4, p = 0.042), but that of prostate cancer does not present a significant difference (χ^2^ = 2.5, p = 0.282).

In pulmonary cancer, the distribution of metastatic lesions in the pelvis of patients with extensive bone metastases was slightly more than that in patients with few and moderate bone metastases (χ^2^ = 6.4, p = 0.042) (Fig 1), but the distributions of bone metastases in the vertebrae (χ^2^ = 1.7, p = 0.433), thoracic bones (χ^2^ = 0.1, p = 0.931), skull (χ^2^ = 1.1, p = 0.565), and extremities (χ^2^ = 4.3, p = 0.119) were not significantly different. Overall, the distributions in different bones of pulmonary cancer were very similar in different lesions.

**Fig 2:**
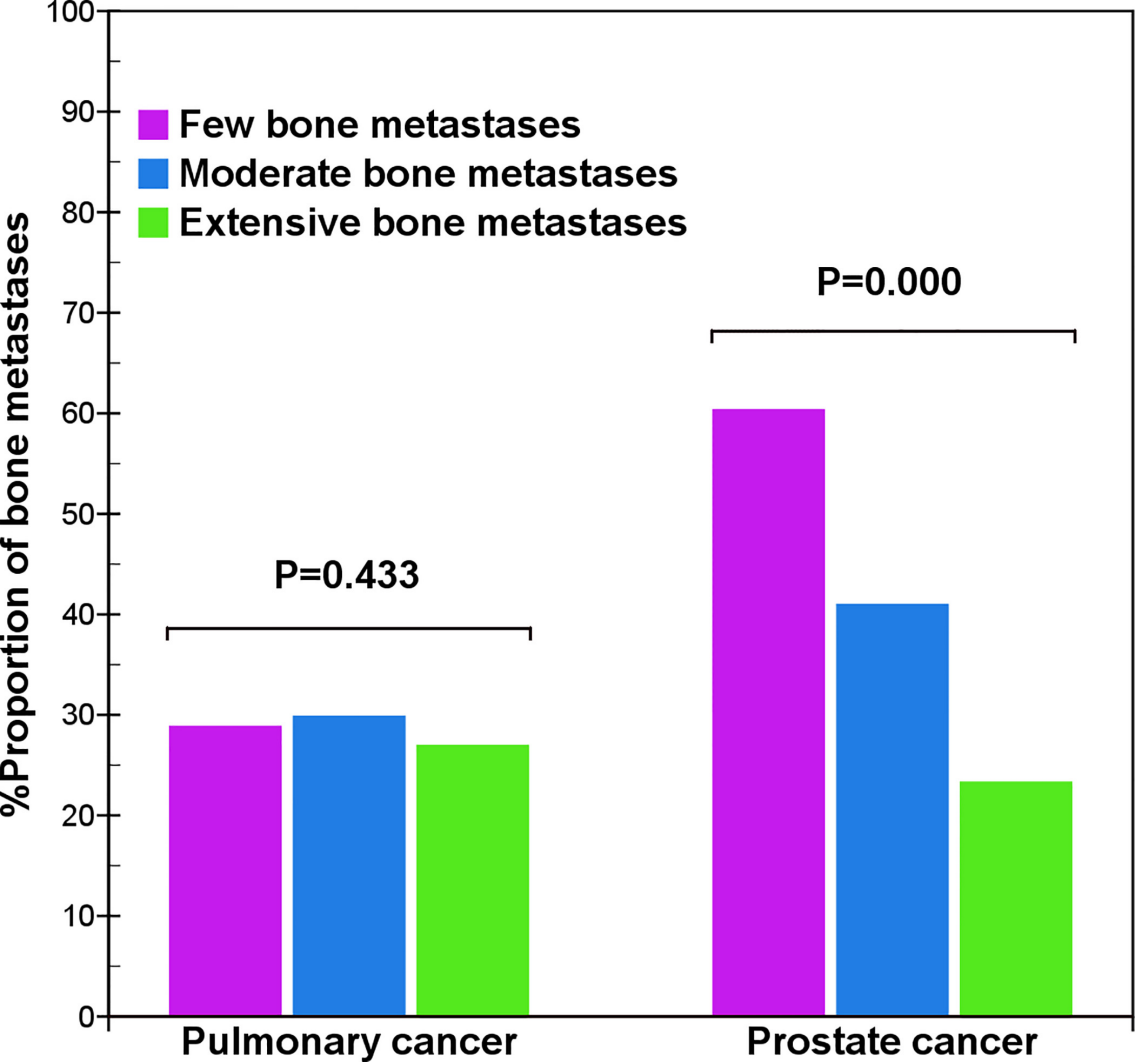
Comparison of vertebral metastases between pulmonary and prostate cancers. The result shows that with an increase of bone metastases, the distribution of vertebral metastases of prostate cancer presents a quickly decreased tendency (χ^2^ = 54.2, p = 0.000), but that of pulmonary cancer does not present a significant difference (χ^2^ = 1.7, p = 0.433).

**Fig 3:**
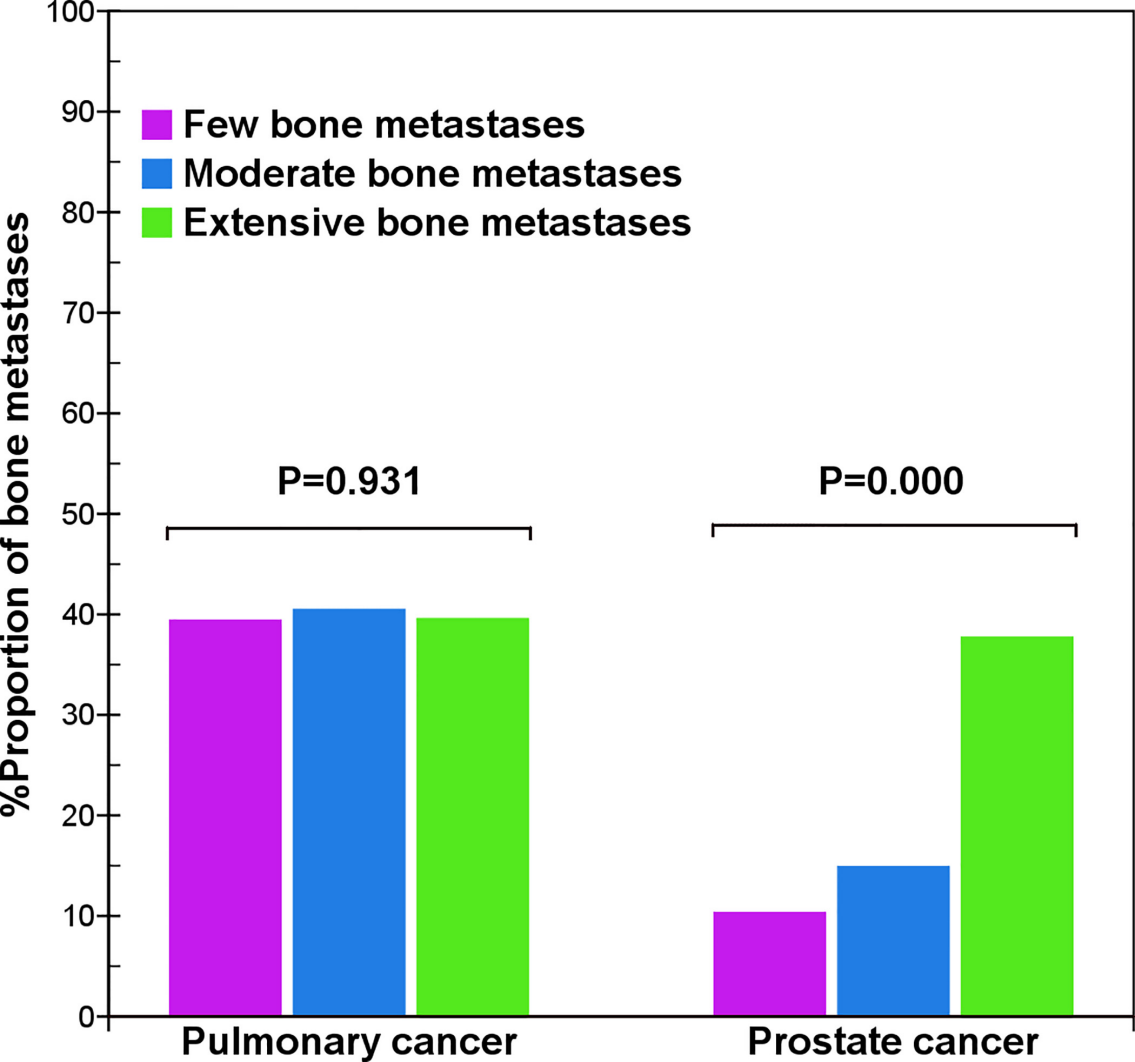
Comparison of thoracic bone metastases between pulmonary and prostate cancers. The result shows that with an increase of bone metastases, the distribution of thoracic bone metastases of prostate cancer presents a gradually increased tendency (χ^2^ = 57.4, p = 0.000), but that of pulmonary cancer does not present a significant difference (χ^2^ = 0.1, p = 0.931).

In prostate cancer, the distribution of bone metastases in the pelvis (χ^2^ = 2.5, p = 0.282), skull (χ^2^ = 3.9, p = 0.141), and extremities (χ^2^ = 0.4, p = 0.808) was not significantly different. However, the distribution was considerably different in the vertebrae (χ^2^ = 54.2, p = 0.000) and thoracic bones (χ^2^ = 57.4, p = 0.000). With an increased total number of bone metastases, there was a decreased tendency to metastasize to the vertebral column (Fig 2), but an increased tendency to spread to the thoracic bones (Fig 3). Therefore, there was a significantly different distribution of metastases in the vertebrae and thoracic bones in prostate cancer, but little difference in distribution in pulmonary cancer.

### 6. Comparison of Bone Metastases in the Vertebrae and Pelvis

**Fig 4:**
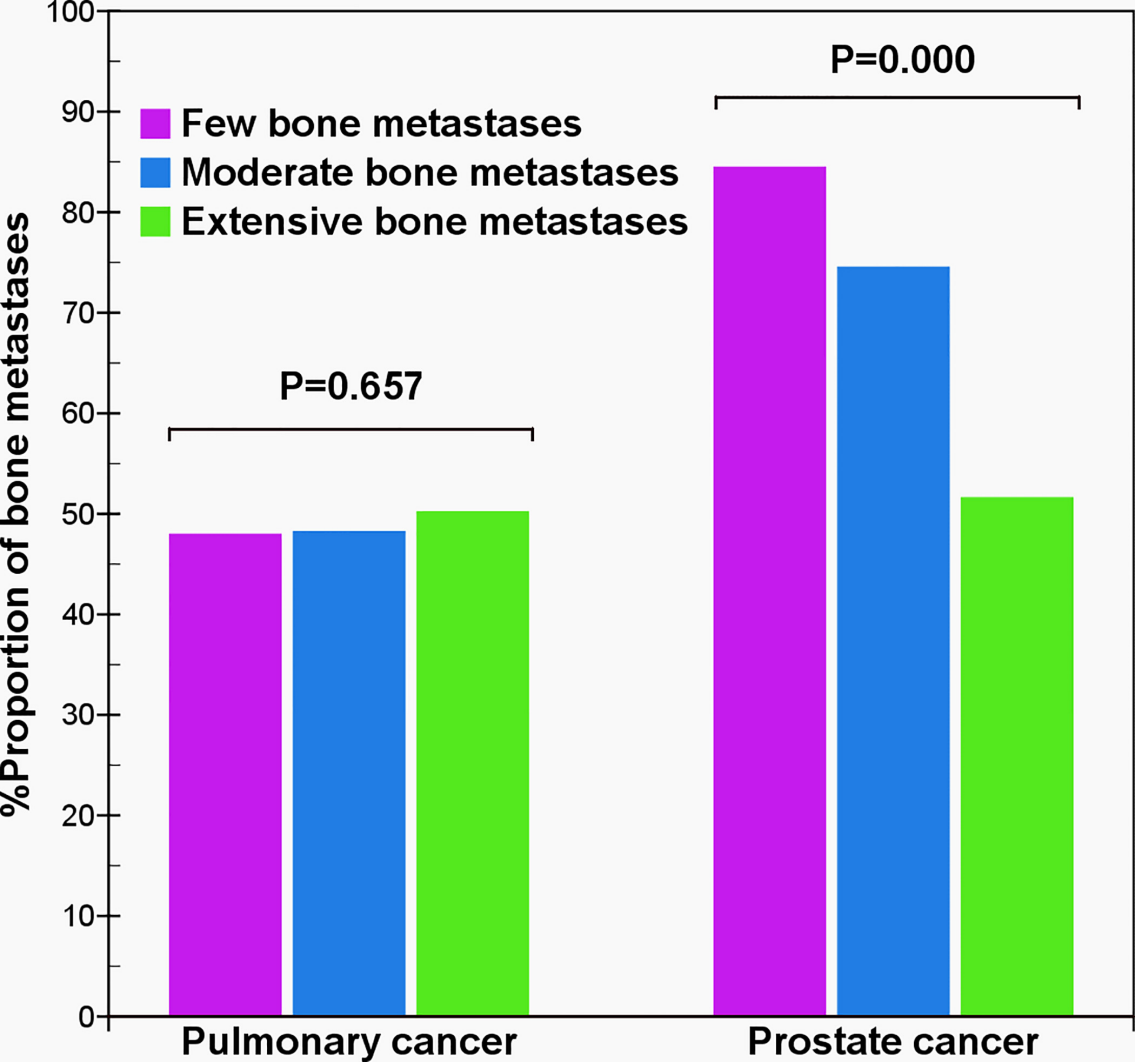
Comparison of vertebra and pelvis metastases between pulmonary and prostate cancers. The result shows that with an increase of bone metastases, the proportion of both of the vertebra and pelvis metastases of prostate cancer presents a gradually decreased tendency (χ^2^ = 57.2, p = 0.000), but that of pulmonary cancer does not present a significant difference (χ^2^ = 0.8, p = 0.657).

In patients with few bone metastases, there was a greater distribution of metastases (84.5%, 49/58) in the vertebrae and pelvis in prostate cancer than (48.0%, 118/246) in pulmonary cancer (χ^2^ = 27.8, p = 0.000). Prostate cancer metastasized to the vertebrae and pelvis more frequently than pulmonary cancer in the early stages of bone metastases, and seldom metastasized to the bones except to the vertebra and pelvis, especially the sternum, collarbone, bladebone, and bones of the upper extremities (S1 Table). When the vertebra and pelvis did not progress into the carcinomatous metastasis, it was very rare for prostate cancer to spread to other bones; however, pulmonary cancer can metastasize to other bones such as the ribs (S1 and S2 Figs) or femurs (S2 Fig). In patients with moderate bone metastases, the distribution (74.5%, 120/161) of prostate cancer metastases was more than that (48.2%, 231/479) of pulmonary cancer (χ^2^ = 35.1, p = 0.000). However, in patients with extensive bone metastases, there was no significant difference in the distribution of metastases in pulmonary (50.2%, 780/1554) and prostate (51.6%, 919/1781) cancers (χ^2^ = 0.7, p = 0.417). Under this condition, cancer cells frequently metastasized to multifarious bones, and bone metastases in pulmonary (S3 Fig) and prostate cancers (S4 Fig) could also mainly distribute in the vertebrae and pelvis. For prostate cancer, the difference in distribution of metastatic lesions in both the vertebrae and pelvis was significantly different (χ^2^ = 57.2, p = 0.000), in contrast to pulmonary cancer (χ^2^ = 0.8, p = 0.657) (Fig 4).

### 7. Comparison of Bone Metastases in Lumbar, Thoracic, and Cervical Vertebrae

The distribution of bone metastases in the lumbar, thoracic, and cervical vertebrae differed in prostate cancer (S2–S4 Tables). In patients with few bone metastases, there was high proportion of metastases in the lumbar vertebrae, with a gradually decreasing tendency to spread from the lumbar and thoracic vertebrae, to the cervical vertebrae (S2 Table). In patients with moderate and extensive bone metastases, the distribution in thoracic vertebrae was more than that in lumbar vertebrae (S3 and S4 Tables). These results indicate that prostate cancer frequently metastasizes to the lumbar vertebrae in the early stages, and then to the thoracic and cervical vertebrae. However, pulmonary cancer more frequently metastasizes to the thoracic vertebrae (S3 and S4 Tables).

## Discussion

Both pulmonary and prostate cancers have a great avidity for bone, frequently leading to painful and untreatable consequences. Patients in the advanced stages of these diseases frequently have bone metastases [24]. Our results showed a high distribution of metastatic lesions in the vertebrae, pelvis, and thoracic bones in pulmonary (89.3%) and prostate cancers (89.5%), demonstrating that these bones are predilection sites of bone metastases in both cancers. These results are in accordance with studies about pulmonary [11–14, 25] and prostate cancers [15–17, 26]. Notwithstanding the small differences in the distribution of bone metastases in the pelvis and thoracic bones between pulmonary and prostate cancers, our results showed that the total distribution of metastatic lesions in the different regional bones were very similar between the two cancer types (Table 1), which is consistent with data from Morgan et al. [3].

Although the spread pathways differ in pulmonary and prostate cancers, our data did not explain the relationship between the different metastatic pathways and the distribution of bone metastases. However, understanding this relationship is very important, as it will allow the detection of primary cancer lesions according to the features of the metastatic lesions, and will allow the differentiation between benign bone lesions and metastatic bone involvement based on the characteristics of known primary tumors. Clearly, the total distributional features of bone metastases in pulmonary and prostate cancers do not aid in the detection of primary tumor lesions or the differentiation of benign and malignant lesions. Batson [1] thought that prostate cancer cells metastasized in the early stages to the pelvis and vertebrae by the vertebral venous system. Bubendorf et al. [2] analyzed the metastatic patterns of prostate cancer in an autopsy study of 1589 patients, and their results strongly supported the metastatic pattern by vertebral veins. However, the results by Morgan et al. [3] and Dodds et al. [4] showed that the differences in the distribution of bone metastases in prostate and pulmonary cancers (or nonprostatic cancer) were not significant; thus, they did not support the concept that the vertebral veins had a unique and substantial role in the dissemination of prostate cancer. Similar to Morgan et al. [3] and Dodds et al. [4], our results on the total distribution of bone metastases in pulmonary and prostate cancer also did not support the role of metastasis by vertebral veins. Nevertheless, our results strongly supported the role of Batson’s vertebral venous plexus in the metastatic patterns of prostate cancer. In this study, in patients with few and moderate bone metastases, the overwhelming majority of bone metastases in prostate cancer were distributed in the vertebrae and pelvis, similar to the distribution feature in prostate cancer of the Batson venous plexus.

Pulmonary and prostate cancers have different distribution features of bone metastases. The distribution of bone metastases in prostate cancer is unstable, in that it is correlated with the total number of bone metastases, and changes as the number of metastases increases, with the most prominent change occurring in the vertebrae and thoracic bones. The proportions of the vertebra and thoracic bone metastases in patients with prostate cancer inversely correlated with the total number of bone metastases, indicating that the distribution of bone metastases in prostate cancer is characterized by preferential metastasis to the vertebrae and then to the thoracic bones. Specifically, in the early stages of prostate cancer, metastases occur in the lumbar vertebrae, followed by the thoracic and cervical vertebrae. The feature of variable distribution of bone metastases in patients with prostate cancer could be interpreted by the carcinomatosis theory of Batson vertebral veins [1]. In this study, the proportion of vertebral metastases in prostate cancer gradually decreased with an increase in the total number of bone metastases. This decrease did not result from a decrease in the actual number of vertebral metastases, but rather, was due to the relatively slow increasing speed of vertebral metastases after many metastatic lesions occupied the vertebrae of limited capacity [23]. In contrast to prostate cancer, the distribution of bone metastases in pulmonary cancer did not significantly change with an increase in the total number of bone metastases. In addition, the proportion of metastatic lesions in each of bone regions was largely similar in different lesions. The distribution feature of bone metastases in pulmonary cancer may be explained by the metastatic pattern of pulmonary veins. Pulmonary cancer cells spread to the bones of different regions with approximately changeless proportions by pulmonary veins. Therefore, the distribution of bone metastases of pulmonary cancer is characterized by the randomness and stability in the proportion of metastatic lesions. In pulmonary and prostate cancers, the different features in the distribution of bone metastases simply result from the different spread pathways.

The early distribution of bone metastases presents great differences between pulmonary and prostate cancers. Our results showed that in the early stage, prostate cancer cells frequently encroach on the vertebrae (especially lumbar vertebrae), but do not frequently metastasize to the thoracic bones. In pulmonary cancer, cells not only frequently encroach on the vertebrae, but also metastasize to the thoracic bones. The difference in the early distribution of bone metastases in pulmonary and prostate cancers is useful for differentiating between benign and malignant bone diseases and for detecting primary tumors. In patients with extensive bone metastases, despite the fact that there were some small differences in the distributions of bone metastases between pulmonary and prostate cancers, the bony distributions of the two tumors were still very similar overall. The similarity manifested in that on one hand, despite the different pathways through which tumor cells disseminated, the final predilection sites of bone metastases of pulmonary and prostate cancer were identical (vertebrae, pelvis, thoracic bones). On the other hand, the distribution of bone metastases could not reflect the difference in the disseminated pathways at this time. Therefore, the difference in the distribution of bone metastases between pulmonary and prostate cancers, which results from the different spread pathways, is not present in patients with extensive bone metastases but occurs in early bone metastases.

## Conclusions

As common malignant tumors, both pulmonary and prostate cancers frequently disseminate to the bones. Our study showed that the distribution features of bone metastases in pulmonary and prostate cancers are different. The distribution of bone metastases in prostate cancer correlated with the total number of bone metastases, and had different features in different lesions. However, the distributions of bone metastases in pulmonary cancer were stable in different lesions. Prostate cancer is characterized by early dissemination to the vertebrae, and then metastasis to the thoracic bones, whereas pulmonary cancer is characterized by random spreading to different bones with the relatively stable proportion. The different features in distribution are mainly present in early bone metastases, not in extensive bone metastases. Our results confirm that the distribution of bone metastases in pulmonary and prostate cancers correlate with their different patterns of hematogenous dissemination. Pulmonary veins and the vertebral vein system separately play important roles in the dissemination of pulmonary and prostate cancers in the early stage of bone metastases.

## Supporting Information

S1 Fig Bone scintigraphy results of a 46-year-old male patient with pulmonary squamous-cell carcinoma. (**A)**, Bone scintigraphy before pneumonectomy. The figure shows no metastatic bony lesion in whole body. (**B)**, Bone scintigraphy at the 8^th^ month after pneumonectomy. The figure shows that an injured rib (white arrow) due to surgical procedures presents the increased uptake. (**C)**, Bone scintigraphy at the 22^nd^ month after pneumonectomy. The figure shows that the radioactive uptake of the injured rib is lower at the 22^nd^ month (white arrow) than at the 8th month, and two rib metastases (green arrow) are detected.
(TIF)

S2 Fig Bone scintigraphy of a 60-year-old male patient with pulmonary adenocarcinoma. The figure shows that bone metastases are distributed in ribs and a femur without the vertebra and pelvis metastasis.
(TIF)

S3 Fig Bone scintigraphy of a 55-year-old male patient with bronchioloalveolar carcinoma. The figure shows that the extensive bone metastases are distributed mainly in the vertebrae and pelvis.
(TIF)

S4 Fig Bone scintigraphy of a 58-year-old male patient with prostate cancer. The figure shows that the extensive bone metastases are distributed mainly in the vertebrae and pelvis.
(TIF)

S1 Table Comparison of total distribution of bone metastases (n = 4279) between pulmonary and prostate cancers. (DOC)

S2 Table Comparison of bone metastases between pulmonary and prostate cancers in patients with few bone metastases (n = 304). (DOC)

S3 Table Comparison of bone metastases between pulmonary and prostate cancers in patients with moderate bone metastases (n = 640). (DOC)

S4 Table Comparison of bone metastases between pulmonary and prostate cancers in patients with extensive bone metastases (n = 3335). (DOC)
