# Supplementary figures and images for: Distribution Features of Skeletal Metastases: A Comparative Study between Pulmonary and Prostate Cancers
Source: PLoS One. 2015 Nov 23;10(11):e0143437. doi: 10.1371/journal.pone.0143437 (PMC4658130; doi:10.1371/journal.pone.0143437)

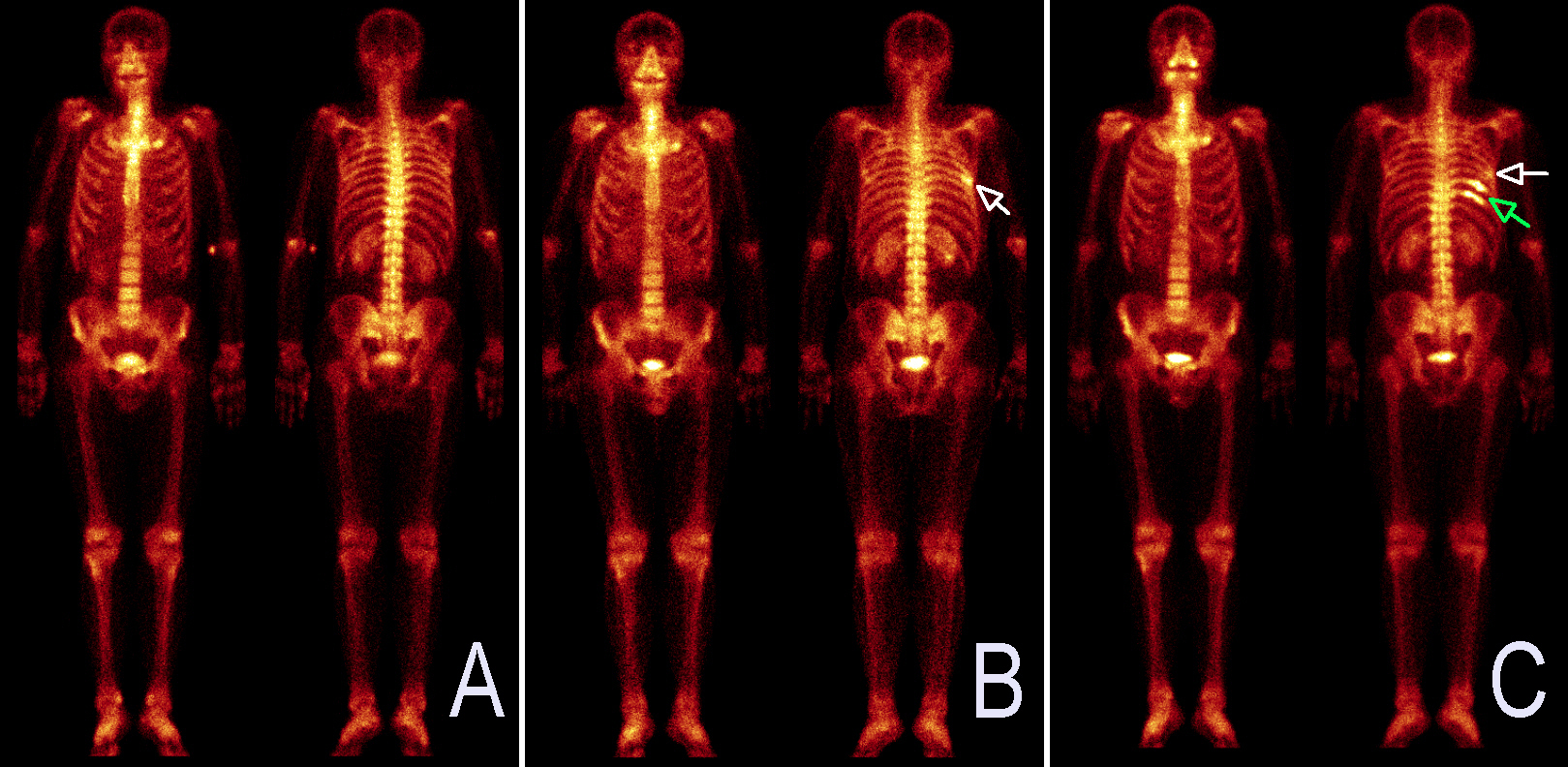

Supplement: S1 Fig — (A), Bone scintigraphy before pneumonectomy. The figure shows no metastatic bony lesion in whole body. (B), Bone scintigraphy at the 8th month after pneumonectomy. The figure shows that an injured rib (white arrow) due to surgical procedures presents the increased uptake. (C), Bone scintigraphy at the 22nd month after pneumonectomy. The figure shows that the radioactive uptake of the injured rib is lower at the 22nd month (white arrow) than at the 8th month, and two rib metastases (green arrow) are detected. (TIF) [file pone.0143437.s001.tif]

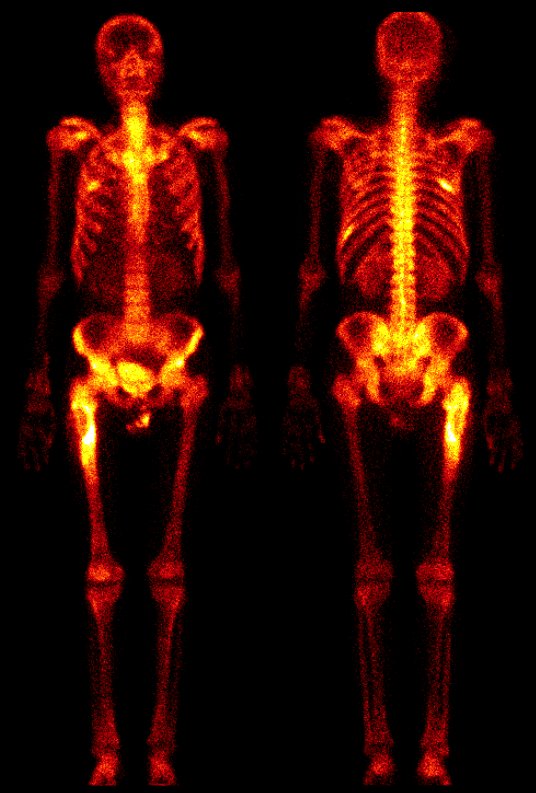

Supplement: S2 Fig — The figure shows that bone metastases are distributed in ribs and a femur without the vertebra and pelvis metastasis. (TIF) [file pone.0143437.s002.tif]

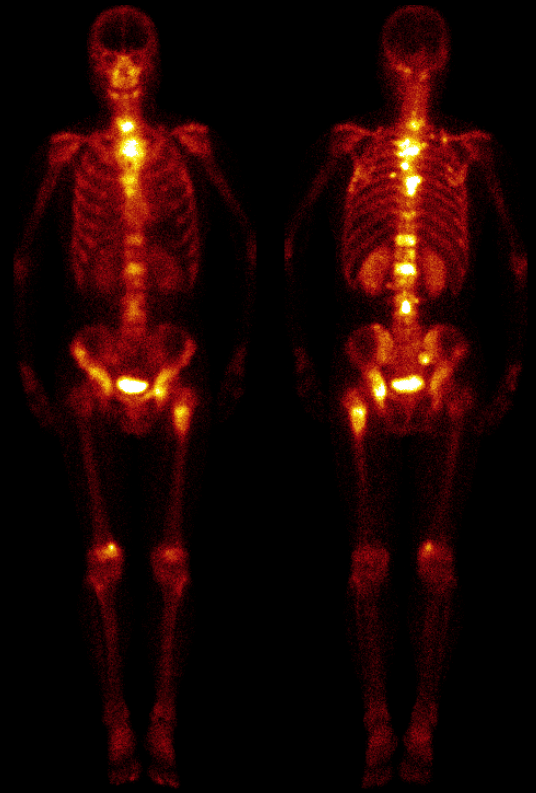

Supplement: S3 Fig — The figure shows that the extensive bone metastases are distributed mainly in the vertebrae and pelvis. (TIF) [file pone.0143437.s003.tif]

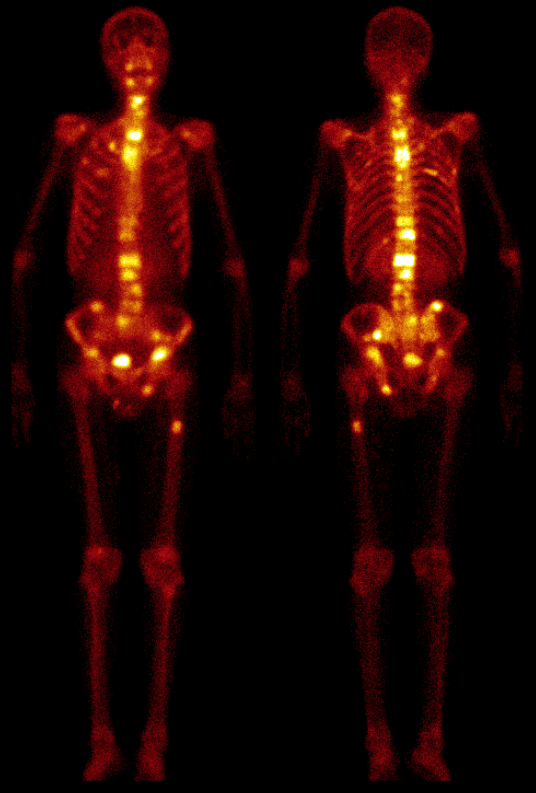

Supplement: S4 Fig — The figure shows that the extensive bone metastases are distributed mainly in the vertebrae and pelvis. (TIF) [file pone.0143437.s004.tif]
