# Supplementary material for: Distribution Features of Skeletal Metastases: A Comparative Study between Pulmonary and Prostate Cancers
Source: PLoS One. 2015 Nov 23;10(11):e0143437. doi: 10.1371/journal.pone.0143437 (PMC4658130; doi:10.1371/journal.pone.0143437)
Supplement: S1 Table — (DOC) [file pone.0143437.s005.doc]

**S1 Table.** **Comparison of total distribution of bone metastases (n=4279) between pulmonary and prostate cancers.**

| **Skeleton** | **Pulmonary cancer (n=2279)** | |  | **Prostate cancer (n=2000)** | | ***χ*^2^** | ***p* value** |
| --- | --- | --- | --- | --- | --- | --- | --- |
|  | **n** | **%** |  | **n** | **%** |  |  |
| **Cervical vertebrae** | 53 | 2.33 |  | 60 | 3.00 | 1.879 | 0.170 |
| **Thoracic vertebrae** | 360 | 15.80 |  | 296 | 14.80 | 0.816 | 0.366 |
| **Lumbar vertebrae** | 220 | 9.65 |  | 160 | 8.00 | 3.616 | 0.057 |
| **Sacrococcyx** | 81 | 3.55 |  | 96 | 4.80 | 4.158 | 0.041 |
| **Ilium** | 262 | 11.50 |  | 276 | 13.80 | 5.132 | 0.023 |
| **Ischium** | 91 | 3.99 |  | 109 | 5.45 | 5.062 | 0.024 |
| **Pubis** | 62 | 2.72 |  | 91 | 4.55 | 10.339 | 0.001 |
| **Ribs** | 729 | 31.99 |  | 578 | 28.90 | 4.794 | 0.029 |
| **Sternum** | 55 | 2.41 |  | 58 | 2.90 | 0.979 | 0.323 |
| **Bladebone** | 94 | 4.12 |  | 44 | 2.20 | 13.001 | 0.000 |
| **Collarbone** | 28 | 1.23 |  | 22 | 1.10 | 0.153 | 0.696 |
| **Skull** | 81 | 3.55 |  | 65 | 3.25 | 0.300 | 0.584 |
| **Bone of upper Extremities** | 43 | 1.89 |  | 42 | 2.10 | 0.248 | 0.618 |
| **Bone of lower Extremities** | 120 | 5.27 |  | 103 | 5.15 | 0.029 | 0.865 |

Note: n, the lesion number of bone metastases. Chi-square test of likelihood ratio was performed to compare the difference of the proportions of bone metastases between pulmonary and prostate cancers.
