# Supplementary material for: Distribution Features of Skeletal Metastases: A Comparative Study between Pulmonary and Prostate Cancers
Source: PLoS One. 2015 Nov 23;10(11):e0143437. doi: 10.1371/journal.pone.0143437 (PMC4658130; doi:10.1371/journal.pone.0143437)
Supplement: S2 Table — (DOC) [file pone.0143437.s006.doc]

**S2 Table. Comparison of bone metastases between pulmonary and prostate cancers in patients with few bone metastases (n=304).**

| **Skeleton** | **Pulmonary cancer (n=246)** | |  | **Prostate cancer (n=58)** | | ***χ*^2^** | ***p* value** |
| --- | --- | --- | --- | --- | --- | --- | --- |
|  | **n** | **%** |  | **n** | **%** |  |  |
| **Cervical vertebrae** | 1 | 0.41 |  | 2 | 3.45 | - | - |
| **Thoracic vertebrae** | 34 | 13.82 |  | 10 | 17.24 | 0.427 | 0.513 |
| **Lumbar vertebrae** | 36 | 14.63 |  | 23 | 39.66 | 16.458 | 0.000 |
| **Sacrococcyx** | 6 | 2.44 |  | 2 | 3.45 | 0.174 | 0.676 |
| **Ilium** | 27 | 10.98 |  | 6 | 10.34 | 0.020 | 0.889 |
| **Ischium** | 10 | 4.06 |  | 2 | 3.45 | 0.049 | 0.825 |
| **Pubis** | 4 | 1.63 |  | 4 | 6.90 | 3.991 | 0.046 |
| **Ribs** | 87 | 35.37 |  | 5 | 8.62 | 19.042 | 0.000 |
| **Sternum** | 3 | 1.22 |  | 1 | 1.72 | 0.086 | 0.769 |
| **Bladebone** | 7 | 2.84 |  | 0 | 0.00 | - | - |
| **Collarbone** | 0 | 0.00 |  | 0 | 0.00 | - | - |
| **Skull** | 6 | 2.44 |  | 0 | 0.00 | - | - |
| **Bone of upper Extremities** | 8 | 3.25 |  | 0 | 0.00 | - | - |
| **Bone of lower Extremities** | 17 | 6.91 |  | 3 | 5.17 | 0.244 | 0.621 |

Note: n, the lesion number of bone metastases. Chi-square test of likelihood ratio was performed to compare the difference of the proportions of bone metastases between pulmonary and prostate cancers.
