# Supplementary material for: Distribution Features of Skeletal Metastases: A Comparative Study between Pulmonary and Prostate Cancers
Source: PLoS One. 2015 Nov 23;10(11):e0143437. doi: 10.1371/journal.pone.0143437 (PMC4658130; doi:10.1371/journal.pone.0143437)
Supplement: S3 Table — (DOC) [file pone.0143437.s007.doc]

**S3 Table. Comparison of bone metastases between pulmonary and prostate cancers in patients with moderate bone metastases (n=640).**

| **Skeleton** | **Pulmonary cancer (n=479)** | |  | **Prostate cancer (n=161)** | | **χ^2^** | ***p* value** |
| --- | --- | --- | --- | --- | --- | --- | --- |
|  | **n** | **%** |  | **n** | **%** |  |  |
| **Cervical vertebrae** | 9 | 1.67 |  | 5 | 3.11 | 1.139 | 0.286 |
| **Thoracic vertebrae** | 83 | 17.33 |  | 39 | 24.22 | 3.569 | 0.059 |
| **Lumbar vertebrae** | 52 | 10.86 |  | 22 | 13.66 | 0.900 | 0.343 |
| **Sacrococcyx** | 11 | 2.30 |  | 14 | 8.70 | 11.240 | 0.001 |
| **Ilium** | 48 | 10.02 |  | 22 | 13.66 | 1.573 | 0.210 |
| **Ischium** | 17 | 3.55 |  | 11 | 6.83 | 2.830 | 0.093 |
| **Pubis** | 12 | 2.51 |  | 7 | 4.35 | 1.309 | 0.252 |
| **Ribs** | 160 | 33.40 |  | 22 | 13.66 | 25.544 | 0.000 |
| **Sternum** | 5 | 1.04 |  | 1 | 0.62 | 0.253 | 0.615 |
| **Bladebone** | 18 | 3.76 |  | 0 | 0.00 | - | - |
| **Collarbone** | 11 | 2.30 |  | 1 | 0.62 | 2.285 | 0.131 |
| **Skull** | 17 | 3.55 |  | 5 | 3.11 | 0.073 | 0.787 |
| **Bone of upper Extremities** | 7 | 1.46 |  | 5 | 3.11 | 1.590 | 0.207 |
| **Bone of lower Extremities** | 30 | 6.26 |  | 7 | 4.35 | 0.860 | 0.354 |

Note: n, the lesion number of bone metastases. Chi-square test of likelihood ratio was performed to compare the difference of the proportions of bone metastases between pulmonary and prostate cancers.
