# Supplementary material for: Distribution Features of Skeletal Metastases: A Comparative Study between Pulmonary and Prostate Cancers
Source: PLoS One. 2015 Nov 23;10(11):e0143437. doi: 10.1371/journal.pone.0143437 (PMC4658130; doi:10.1371/journal.pone.0143437)
Supplement: S4 Table — (DOC) [file pone.0143437.s008.doc]

**S4 Table.** **Comparison of bone metastases between pulmonary and prostate cancers in patients with extensive bone metastases (n=3335).**

| **Skeleton** | **Pulmonary cancer (n=1554)** | |  | **Prostate cancer (n=1781)** | | ***χ*^2^** | ***p* value** |
| --- | --- | --- | --- | --- | --- | --- | --- |
|  | **n** | **%** |  | **n** | **%** |  |  |
| **Cervical vertebrae** | 44 | 2.83 |  | 53 | 2.98 | 0.061 | 0.804 |
| **Thoracic vertebrae** | 243 | 15.64 |  | 247 | 13.87 | 2.067 | 0.151 |
| **Lumbar vertebrae** | 132 | 8.49 |  | 115 | 6.46 | 5.008 | 0.025 |
| **Sacrococcyx** | 64 | 4.12 |  | 80 | 4.49 | 0.281 | 0.596 |
| **Ilium** | 187 | 12.03 |  | 248 | 13.92 | 2.627 | 0.105 |
| **Ischium** | 64 | 4.12 |  | 96 | 5.39 | 2.964 | 0.085 |
| **Pubis** | 46 | 2.96 |  | 80 | 4.49 | 5.441 | 0.020 |
| **Ribs** | 482 | 31.02 |  | 551 | 30.94 | 0.002 | 0.961 |
| **Sternum** | 47 | 3.02 |  | 56 | 3.14 | 0.040 | 0.842 |
| **Bladebone** | 69 | 4.44 |  | 44 | 2.47 | 9.844 | 0.002 |
| **Collarbone** | 17 | 1.09 |  | 21 | 1.18 | 0.054 | 0.817 |
| **Skull** | 58 | 3.73 |  | 60 | 3.37 | 0.321 | 0.571 |
| **Bone of upper Extremities** | 28 | 1.80 |  | 37 | 2.08 | 0.331 | 0.565 |
| **Bone of lower Extremities** | 73 | 4.70 |  | 93 | 5.22 | 0.484 | 0.487 |

Note: n, the lesion number of bone metastases. Chi-square test of likelihood ratio was performed to compare the difference of the proportions of bone metastases between pulmonary and prostate cancers.
